# Building social capital through breastfeeding peer support: insights from an evaluation of a voluntary breastfeeding peer support service in North-West England

**DOI:** 10.1186/s13006-015-0039-4

**Published:** 2015-04-02

**Authors:** Gill Thomson, Marie-Clare Balaam, Kirsty Hymers

**Affiliations:** Maternal and Infant Nutrition and Nurture Unit, University of Central Lancashire, Preston, Lancashire PR1 2HE UK; Research in Childbirth and Health Unit, University of Central Lancashire, Preston, Lancashire PR1 2HE UK; East Lancashire Hospitals NHS Trust, Royal Blackburn Hospital, Haslingden Road, Blackburn, Lancashire BB2 3HH UK

**Keywords:** Breastfeeding peer support, Voluntary, Social capital, Qualitative, Women, Professionals

## Abstract

**Background:**

Peer support is reported to be a key method to help build social capital in communities. To date there are no studies that describe how this can be achieved through a breastfeeding peer support service. In this paper we present findings from an evaluation of a voluntary model of breastfeeding peer support in North-West England to describe how the service was operationalized and embedded into the community. This study was undertaken from May, 2012 to May, 2013.

**Methods:**

Interviews (group or individual) were held with 87 participants: 24 breastfeeding women, 13 peer supporters and 50 health and community professionals. The data contained within 23 monthly monitoring reports (January, 2011 to February 2013) compiled by the voluntary peer support service were also extracted and analysed.

**Results:**

Thematic analysis was undertaken using social capital concepts as a theoretical lens. Key findings were identified to resonate with’bonding’, ‘bridging’ and ‘linking’ forms of social capital. These insights illuminate how the peer support service facilitates ‘bonds’ with its members, and within and between women who access the service; how the service ‘bridges’ with individuals from different interests and backgrounds, and how ‘links’ were forged with those in authority to gain access and reach to women and to promote a breastfeeding culture. Some of the tensions highlighted within the social capital literature were also identified.

**Conclusions:**

Horizontal and vertical relationships forged between the peer support service and community members enabled peer support to be embedded into care pathways, helped to promote positive attitudes to breastfeeding and to disseminate knowledge and maximise reach for breastfeeding support across the community. Further effort to engage with those of different ethnic backgrounds and to resolve tensions between peer supporters and health professionals is warranted.

## Introduction

Breastfeeding peer support was initially developed in the 1950’s in America through the formation of the La Leche League [1]. Since this time, breastfeeding peer support services have grown exponentially through international and national organisations as well as via local ‘home grown’ services. A concept analysis of peer support undertaken by Cindy-Lee Dennis provides the following definition:

*‘The provision of emotional, appraisal, and informational assistance by a created social network member who possesses experiential knowledge of a specific behaviour or stressor and similar characteristics as the target population*.’ ([2] p.329)*.*

Breastfeeding peer support can be offered via face to face contacts (in home, hospital or community locations such as via breastfeeding groups), telephone, Short Message Service (SMS) or social media, e.g. Facebook groups. While a key premise of breastfeeding peer support is to help women to sustain self-feeding methods, it is also perceived to be a key method to address cultural barriers [3-6] through peer supporters operating as positive breastfeeding role models [3,4,7-9].

A recently updated Cochrane review of interventions to increase duration of breastfeeding [10] reported that skilled support, peer or professional, proactively offered to women who want to breastfeed can increase breastfeeding duration rates. However, a recent meta-regression of 17 breastfeeding peer support Randomised Controlled Trials (RCTs) reported that while breastfeeding peer support had a positive impact on breastfeeding duration rates in low and middle income countries, it had less impact in high-income countries (particularly the UK) [11]. These findings appear contrary to insights generated from qualitative research. While qualitative research is criticised for its lack of generalisability and potential for bias, this approach is valued for its in-depth insights into attitudes, feelings and behaviours [12]. For example, a review of 79 UK breastfeeding practice reports undertaken by Dykes [3,4] and other UK based qualitative research [9,13] suggest that peer support is highly valued by women and can influence women’s confidence and self-efficacy to breastfeed. Hoddinott *et al.* [14] and Thomson & Trickey [15] also question the validity of the breastfeeding peer support RCTs due to the wide heterogeneity in the trial design, implementation, contextual issues and outcomes of the trial data.

Breastfeeding peer support is currently advocated in health policy as a way of promoting equality of access to health care through using a community-oriented, health promotional approach [16]. Lay or peer support interventions are also recommended in various national and international guidelines and policy documents to help increase breastfeeding rates [17-20]. Within the recent UK National Institute of Health and Clinical Excellence commissioning guidelines for breastfeeding peer support provision, the outcomes of peer support relate to increasing the number of women who initiate and continue to breastfeed as well as the potential of this approach to reduce inequalities and build capacity within local communities [19]. Community members are believed to be best placed to promote and support the healthier life choices of others within their community due to their innate understanding of the social realities of everyday life [21]. Although, the potential for peer support to create negative outcomes, such as instilling guilt and pressure through attempts to alter an individual’s behaviour should also be acknowledged [22].

Over the last few decades attention has turned towards the importance and influence of social capital. While the term *capital* generally refers to resources, social scientists, epidemiologists and health economists argue that individuals and communities require access to a range of resources to enjoy healthy, productive relationships in safe, sustainable environments [23-25]. The first systematic contemporary analysis of social capital undertaken by Pierre Bourdieu identified that it included two dimensions - social networks and connections/relationships and sociability [26]. Bourdieu considered that the action of these social networks operated to increase the social capital available to some individuals or groups of individuals through the exclusion of others [26]. A later definition offered by Putman offers a different and more inclusive perspective by perceiving social capital as being collectively produced through shared norms, collective association and trust [27].

Social capital is considered to be an ecological based concept, with the term ‘ecological’ being defined by Hofmeyer & Marck as a *‘living system’s capacity to function effectively and resourcefully support its inhabitants’* ([25], p.146). Social capital relates to how network connections are mobilised within and between horizontal and vertical based relationships [28]. It concerns social networks that ‘*bond’* similar people and ‘*bridge’* between diverse people with norms of reciprocity [29,30] and considers how trust and tolerance, connectedness, collective action and solidarity are formed and maintained among groups in societies [31,32]. Participation in local community activities has been identified to strengthen social cohesion [33,34], empower individuals, promote further community involvement and concern for others within the community, [34,35] and has the potential to reduce health inequities [35-38].

Peer support is considered to be an important means of creating social capital, through *‘individuals and collectives who care about their own and others’ wellbeing’* ([39], p.119). Positive health and wellbeing outcomes associated with peer support relate to increased access to information, knowledge, competencies and development of meaningful social networks [38,40-42]. The influence and value of peer support in terms of engendering social capital have been suggested within a health [43], educational and/or employment context [44,45] and for individuals who are vulnerable or marginalised, i.e. mental health [46], disability [47] or experience social isolation [48]. While research into breastfeeding peer support has tended to focus on its effectiveness (e.g. [11]) or value for women (e.g. [9,13]), to date there are no studies that consider how breastfeeding peer support can help to build social capital. Insights into how breastfeeding peer support engenders social capital may well help to identify key methods through which breastfeeding can be promoted and sustained.

In this paper we report on qualitative and monitoring data from an evaluation of a voluntary breastfeeding peer support service in North-West England. Through drawing on social capital concepts we describe and consider how the peer support service has created horizontal and vertical relationships to provide awareness, information and maximise reach for breastfeeding support across the community.

## Methods

### Study context

The Breastfeeding Network (BfN) is a UK national voluntary breastfeeding organisation that provides breastfeeding training to local peer supporters and breastfeeding support to new mothers predominantly via breastfeeding groups and breastfeeding helplines (National Breastfeeding Helpline and BfN Supporterline). In 2008, as part of the wider Baby Friendly Initiative Community implementation in Blackpool, the BfN were commissioned to provide a systematic, targeted and comprehensive breastfeeding peer support service across the antenatal, hospital and postnatal period (up to 8 weeks) named the Star Buddies service. In 2009, a similar service was commissioned in the North Lancashire area on a more restricted basis. Antenatal and hospital bedside support is provided to all mothers, however the comprehensive eight week programme of community support is only offered to mothers residing within three geographical areas that have the lowest breastfeeding continuation rates. Nineteen paid peer supporters (who all work on a part-time basis) together with support from a large body of voluntary peer supporters provide the comprehensive service in Blackpool and North Lancashire.

To support the needs of other mothers residing in North Lancashire and to test out a new model of voluntary breastfeeding support, a more flexible, needs-led service was developed by the BfN. The rationale for this service was based on feedback collected by the BfN that volunteers a) can feel isolated post-training, b) were not always providing support that suited their needs, skills or confidence level, c) feel overwhelmed in undertaking prerequisite governance checks and d) there was a need to ensure that BfN mandatory training requirements were adhered to. These issues were believed to impact on volunteer retention rates and limited supporter hours being provided. A more coordinated volunteer service was therefore developed through the appointment of six paid Volunteer Coordinators (VCs) (all of whom work on a part-time basis) who had responsibility to coordinate volunteers and services within different localities in North Lancashire. The number of voluntary peer supporters’ active each month across all the six areas ranged from 25–53. In addition, the paid peer supporters (n = 8) who were employed to deliver the comprehensive North Lancashire commissioned service also provided volunteer hours to support this work. The key roles of the VCs as defined by the service were to a) work with localities and mothers to tailor the support offered by volunteers; b) collect and record volunteer activity (discussed in more depth below); c) help volunteers comply with mandatory training after they qualify; d) arrange prompt payment of expenses; e) help coordinate training events; f) arrange social and networking events and g) thank the volunteers.

An overview of where and how voluntary breastfeeding peer support is provided across the perinatal period is detailed in Table 1.

**Table 1:** **Overview of where and how breastfeeding peer support is provided across the perinatal period**

**Antenatal**	**Intrapartum**	**Postnatal**
Parent education classes	Antenatal/postnatal wards	Breastfeeding groups
Breastfeeding groups	Neonatal Unit	Home visits
Antenatal clinics		Telephone/SMS
Breastfeeding helpline(s)		Postnatal clinics
		Various mother and baby groups/activities
		Breastfeeding helpline(s)

The area covered by NHS North Lancashire at the time of undertaking this evaluation included the rural and coastal areas of Fylde and Wyre and the larger urban centres of Lancaster, Morecambe and Fleetwood. The North Lancashire area has a mixed socio-economic profile with several wards in Morecambe and Fleetwood rated amongst the most deprived in the North West [49]. The 2011 census showed that the largest ethnic group in the area covered by NHS North Lancashire was White British (95-98%) with a lower than national average Black Minority Ethnic (BME) population (4.4-1.8%) [50].

All paid Star Buddies have undertaken the Open College Network (OCN) Breastfeeding Helpers (6 or 12 weeks) and the more advanced Breastfeeding Supporters (12 months) accredited courses provided by the BfN. The Breastfeeding Helpers course covers the basics of breastfeeding management, reflection on personal experience, listening skills, working in a group, role of BfN and other agencies working with new mothers, breastfeeding support, sources of breastfeeding information, role of research and the need to protect infant feeding from commercial interests. The Breastfeeding Supporters course develops the peer supporter’s knowledge and skills so she can work independently and take calls on the breastfeeding helplines (National Breastfeeding Helpline and BfN Supporterline). While voluntary peer supporters have to undertake the Breastfeeding Helper course to become registered with the BfN, they are also encouraged to access the Supporters course.

All peer supporters sign up to a BfN Code of Conduct which sets out the standards for the organisation. BfN also requires that all peer supporters receive regular supervision to allow for reflective practice and development. All BfN volunteers have to complete mandatory training on areas such as hand hygiene, safeguarding and information governance. The peer supporters are also encouraged (and provided with funds) to attend the BfN’s Annual General Meeting. In addition and where possible, volunteers are able to attend local and national breastfeeding related events, e.g. the annual UNICEF Baby Friendly Initiative Conference.

**Figure 1:**
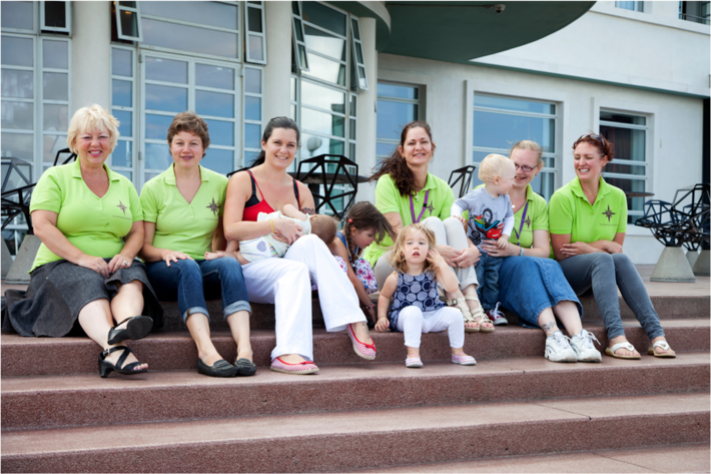
**Paid and voluntary Star Buddies peer supporters.**

**Figure 2:**
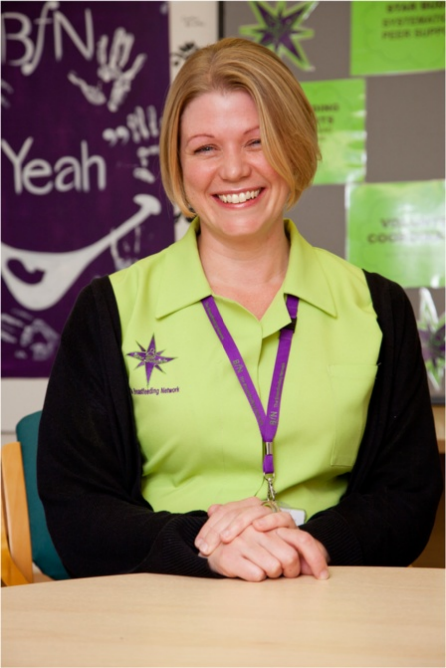
**Community Star Buddies peer supporter.**

**Figure 3:**
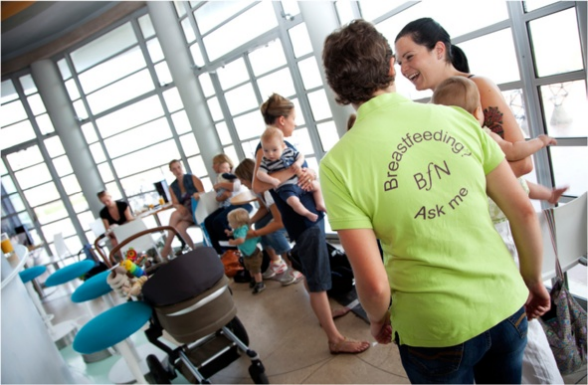
**Star Buddies peer supporter and breastfeeding women at breastfeeding event.**

All the Star Buddies (paid and voluntary) wear a ‘uniform’ which comprises a green T-Shirt with the Star Buddies logo on the front, and the BfN logo and the words ‘Breastfeeding’ and ‘Ask Me’ displayed on the back (see Figures 1, 2 and 3).

Research staff from the University of Central Lancashire were commissioned to undertake an exploratory study to uncover how the voluntary breastfeeding peer support service was being operationalised in North Lancashire. This study was undertaken from May, 2012 – May, 2013 and comprised of interviews (group or individual) and a review of monthly monitoring service data.

### Participants & recruitment

Following management approval, health and community professionals were approached via email to take part in the study. Peers supporters were recruited via the VCs and breastfeeding mothers who had accessed the peer support service were recruited by the VCs and voluntary peer supporters. In all occasions, participants were issued with an information sheet and asked to contact the evaluation team direct if they wanted to participate.

A total of 87 participants took part in semi-structured group (n = 9) or individual face to face or telephone interviews (n = 49). Interviews were undertaken with all the VCs (n = 6), seven voluntary peer supporters (22 in total were approached to take part representing a response rate of 32%), 24 breastfeeding women and 50 professionals (health and community). An additional group interview with all the VCs was also undertaken towards the end of the evaluation. An overview of all the participants who took part in this study is detailed in Table 2 and more detailed demographics on the breastfeeding women who participated is presented in Table 3.

**Table 2:** **Overview of participants**

**Type of participant**	**N = 87**
Star buddies volunteer coordinators	6
Voluntary breastfeeding peer supporters	7
Breastfeeding women	24
Midwifery staff	14
Health visitors	11
NHS commissioner	1
Infant feeding co-ordinator/lactation consultant	3
Children’s centre staff^1^	21

^**1**^Children’s Centres were first developed in 1998 under the Sure Start Local Programmes agenda, and were designed to give children ‘the best possible start in life’ by improving childcare, health and family support services available. While originally only situated in high areas of deprivation, from 2002–2012, they were developed in other localities across the UK. Children’s Centres are local community centres which offer a wide range of practical, educational and emotional and health based services for families who have children under the age of five.

**Table 3:** **Socio-demographic characteristics of women participants**

**Characteristics**	**N = 24**
*Age of mothers*	Range 19-47
›20	n = 1
20-29 years	n = 12
30-39 years	n = 9
40 +	n = 2
*Number of children*	
1 child	n = 12
2 children	n = 9
3 children	n = 1
4 children	n = 2
*Age of infants at time of interview*	11 weeks – 8 months
*Ethnicity/nationality*	
White British/European	n = 23
Latin American	n = 1

While the wording of the interview schedules varied by participant group, the key issues addressed concerned the participant’s attitudes and experiences of the peer support service, barriers and facilitators to peer support provision, benefits of peer support and recommendations for improvement.

All interviews took between ~20-60 minutes to complete, were digitally recorded and transcribed in full for analysis purposes.

### Monitoring data

Detailed monthly reports were produced by the voluntary breastfeeding peer support service. Each month the VCs contacted all the volunteers working in their respective area to collect information on the numbers of women supported, as well as where and how this support was provided. All the data were then collated into a monthly report by one of the VCs. While these reports were originally started as a means of monitoring service activities, over time they have become a more standardised reporting tool designed to: promote awareness about various peer support activities within the localities; to collate detailed information on the contacts (types of and numbers) provided by the volunteer peer supporters; to highlight any concerns that have been raised within practice and implications of such, and sharing good practice. The VCs were appointed in September 2010 and data collection on volunteer activities commenced in November, 2010. During the evaluation, all the data detailed within the January, 2011 to February 2013 (n = 23) monthly reports were extracted and analysed.

### Data analysis

Descriptive data from the monthly reports (in terms of numbers and types of contacts provided by the volunteer service) were analysed using Excel. All transcribed data and qualitative data extracted from the monthly reports were entered into a qualitative software package (MAXQDA). Data were subsequently analysed into themes and sub-themes using the method described by Braun & Clark [51]. This process involved reading and re-reading of the transcripts to enable familiarisation; organising and mapping data into meaningful groups or networks; re-reading to ensure accuracy and authenticity, with re-organising and refinement undertaken as appropriate [51].

From the emergent findings, and in order to help understand how the service was being delivered and disseminated across the community, social capital concepts were considered when analysing the data set. Following numerous iterations of reading the social capital literature and transcripts and on-going discussions between GT and MCB, the ‘bonding’, ‘bridging’ and ‘linking’ forms of social capital were found to resonate most closely with the data set. These social capital concepts enabled us to highlight how the peer support service worked within and between the structural forces of their community to create awareness, knowledge, resources, trust and reciprocity, as well as highlighting any difficulties or limitations experienced.

### Ethics

Ethics approval was obtained from NHS Research & Development Units at the participating NHS trust and the BuSH (built environment, sport and health) University ethics sub-committee at the main author’s institute (ethics number 061). Issues of informed consent, confidentiality, withdrawal and anonymity were adhered to throughout.

## Results

Qualitative as well as descriptive data from the monthly reports are reported under the themes of ‘bonding’, ‘bridging’ and ‘linking’ social capital. A selection of quotes have been utilised to illuminate the issues being raised, together with the code HP (to refer to a maternity or health visiting professional), CP (to refer to a community professional, e.g. Children’s Centre Workers), P (to refer to the infant feeding coordinator, lactation consultants or commissioner), PS (to refer to VCs/peer supporter) and M (mother). The term ‘participant’ is used to refer to insights generated across the different participant groups. In the occasions where the findings relate to a particular group, this has been made explicit in the text.

### Bonding social capital

Bonding social capital typically refers to close connections and strong bonds between individuals in closed networks to, *‘bring together people who are like one another in important respects’* ([52] p.11).

Bonding social capital was evident within the peer support service through the regular communication and opportunities for social occasions amongst all members of the service. All VCs contacted their assigned volunteers on a monthly basis to collect data for the monthly reports, to provide information on local activities or training events and to provide on-going and informal supervision. Volunteers had the mobile number of the VCs and were encouraged to contact them when needed. The peer support service also had an active Facebook account where issues and events were shared. Mentoring and support was provided by VCs when volunteers were undertaking new tasks, for example providing shadowing opportunities for those who wished to run breastfeeding groups or offer support in the neonatal unit. At Christmas, all volunteers were bought a gift and/or invited to attend a social event to say ‘thank you’; with picnics and other social occasions (involving wider family members) organised on a more ad-hoc basis. While data on the number of volunteers involved in these activities were not recorded, these insights indicate that the service had created a range of formal and informal connections and support between its members. The positive value placed on these networks and personal contacts was highlighted by the volunteers, ‘*makes you feel like you’re connected to something’* and the VCs:

*We do have get togethers, so they can all meet up, just like friends really and let them know as well that they are appreciated.* (PS_1)

In order to maintain the volunteers’ ‘bond’ with the peer support service, on-going assessments were undertaken between the VCs and the individual peer supporters to ensure that their skills, interests and capacity was suitably matched to their volunteering roles. For example, some volunteers worked on the breastfeeding helpline(s) as this suited their availability, whereas others chose to work directly with women, or in groups at times that fitted with their childcare needs or other work commitments:

*And I could volunteer in the evening to go on the ward but evenings, I was just a bit of a washout really. I thought, I’m not going to be any good talking to a mum who’s all emotional because I’m feeling like that myself…so I’ve kind of been somebody who the Coordinator could* [say] *can I phone you if I’ve got too many people to phone and can you do some phone support? And I’ve been very happy to do that.* (PS_11)

Another aspect of bonding social capital was evident in the relationships forged between women and peer supporters. The peer support service was set up to provide proactive on-going contacts between women and peers across the peri-natal period, e.g. antenatal clinics, the postnatal ward and in the community. Early opportunities for contact were perceived to be important by the participants to provide reassurance, ‘*knowing that there was going to be support there’* and to build connections and familiarity with women and their families:

*I know the Star Buddies are trying to build up the antenatal, so they do have contact with women antenatally, which does seem really positive, getting to know somebody before they’ve even made the decision how they’re going to feed their baby.* (HP_2)

According to Woolcock and Sweetser [53] bonding social capital relates to connections with individuals who are *‘like you’* (p. 26). This was evident in this study as women reported how the peer supporters were mothers *‘like them’* which enabled connections through shared understanding and experiences. The women explained that the supporters *‘know what they’re talking about because they’ve done it themselves’.* They also highlighted the personal qualities of the peer supporters, characterising them as reliable, *‘dedicated to what they do’,*’*enthusiastic’, ‘good at talking to people’, ‘friendly’* and ‘*approachable’.* It was these qualities and what women identified to be the *‘time’* and *‘reassurance’* offered by the peer supporters as well as the *‘non-judgemental’* and flexible based tailored support they received that enabled a ‘trust’ based relationship to be formed. This was commonly expressed by women in terms of the safety and reliability of *‘knowing that I could phone somebody’* or ‘*just having someone there’* when they needed it most.

Breastfeeding groups were offered in all locality areas (n = 6) with a total of 1,011 group sessions run over the evaluation period, supported by 2,429 volunteer hours. Attendance at the groups was generally high, and the peer supporters attributed this success to practical and psychological barriers to access being addressed. For example, crèche facilities were provided at the groups for older children and peer supporters would meet and accompany women to the groups. This thereby provided women with the reassurance of a *‘familiar face’* in an unfamiliar setting. Individuals from across the participant groups emphasised how the breastfeeding groups played an invaluable role in linking and bonding new mothers to each other, thereby creating new and significant social contacts;*’I’ve met some really good lifelong friends’*. Women were also identified to have formed their own social networks outside of the group environment:

*There’s a group of mums now, they keep accessing the sensory room together, but they’ve met in the centre* [breastfeeding group]*.* (CP_12)

Bonding social capital is believed to enable individuals to come together to collectively resolve problems and achieve outcomes of mutual benefit [54]. This was evident in the mother-to-mother relationships that provided reassurance and support from those who had had *‘the same problems’* and which in turn helped to increase the women’s motivation, confidence and capacities to resolve their own breastfeeding challenges; ‘*if it wasn’t for the group I wouldn’t have carried on feeding’*:

*Because to start off with I didn’t know how long I was going to last for, it was hard work, you were up all night, it wasn’t as easy as what you thought it was going to be. So I spoke to those other mums that had done twelve months and you just thought, yes there is other mums out there that breastfeed for a long time.* (M_1)

While bonding social capital is generally perceived to be more apparent within homogenous groups [54], some professionals highlighted how the service had created new communities of breastfeeding women which crossed previous social boundaries:

*Its social groups as well that you wouldn’t, they would never have put themselves with each other. And the little group that comes to X, they’re the most diverse group of ladies, girls whatever, four of them there are. You would never have put them as friends out of there, but it’s because they’ve all got this natural link*. (CP_12)

Bonds that are forged between individuals in a community can lead to mutually beneficial collective action [55]. In this study, some of the women reported how their positive experiences of peer support had fuelled a desire to provide the same for others:

*I wanted to give something back because she’s* [peer supporter] *been really good. So she’s put me in touch, I’m now on the course to do the* [peer support training] *for the Breastfeeding Network* (M_1)

### Bridging social capital

Bridging social capital concerns more distant connections between people, and relates to weaker ties across individuals at a same level of hierarchy, such as those from different interests or backgrounds [56,57]. In this theme we report on how the peer supporters forged links with those from different ethnic, economic and professional backgrounds. As well as how ‘*being known’* in the community led to a diffusion and dissemination of breastfeeding information and support across the community.

Bridging social capital was apparent through the service actively recruiting volunteers from Eastern European (e.g. Bulgarian, Czechoslovakian and Polish) and Spanish backgrounds and engaging with outreach workers to provide support to women from different ethnic backgrounds:

*Well luckily there is a volunteer from that community* [Polish] *so we can use her when we need* [. . .]*. There’s also a large population that have just arrived from Eastern Europe* [. . .] *and there’s a whole load that have come over from Hungary, Czechoslovakia, that area. And again, we employ a Czechoslovakian girl in our staff, and she set up a group for Eastern European families.* (PS_2)

However, some on-going challenges in reaching certain ethnic groups were reported:

*Chinese really stick to themselves and don’t really want the support, so you can’t really get in there. And other Asians, again it’s difficult, they don’t tend to come out the house anyway and they always have their sisters and mothers in their own group. They’re always invited to* [breastfeeding groups] *but it’s a bit difficult to draw them in.* (PS_1)

Engagement with Children’s Centre staff, meant that the peer supporters were able to build links with ‘hard to reach’ families and young mothers who are known to be those who are less likely to breastfeed [58]. For example, volunteers attended Young Parents groups at the Children’s Centres and provided support at a supported accommodation centre for homeless young people and families. While these relationships may not equate to the ‘bonding’ social capital created amongst women who actively engaged with peer support, the service had created opportunities to connect with the more marginalised populations in the community.

The opportunity to form links in order to identify and understand cultural differences in health behaviours was considered essential within the peer support service; ‘*they* [Eastern European women] *believe smoking is OK but they won’t smoke and breastfeed’.* However, many of the peer supporters emphasised a need for on-going communication with local women as well as professionals to ensure that their service was appropriate and responsive for all those residing within the community:

*You have to find what works with the people you’re working with and everybody’s different, every area is different.* […]. *Because as the years go by people change and how they want it changes, so it’s keeping on top of that.* (PS_2)

Bridging social capital involves connections between individuals who are ‘*not like themselves’* (e.g. non breastfeeding mothers) to open up new opportunities, span social boundaries and provide connections to their current networks [56,57,59]. As wider social networks are known to have a significant impact on women’s decisions and experiences of infant feeding [58], the peer support service involved partners and other family members in antenatal workshops, breastfeeding groups as well as during home visits. Peer supporters explained the need to include ‘*their* [the woman’s] *support system,*’ as they *‘all need to understand how it works and what’s going to happen and what’s going on and why it’s a good thing’* so that they can best support the mother. The peer supporters reported how they took *‘every opportunity’* to engage with fathers and wider social networks through encouraging ‘*mums to bring grandmas’* and *‘getting as many of the family’* involved because ‘*that makes a huge difference’*. A grandmother peer support training course was provided in one of the localities. Breastfeeding groups also coincided with antenatal clinics in community locations. This was perceived as important to enable pregnant women to observe breastfeeding and promote their self-beliefs and self-efficacy for breastfeeding, and corresponds with the ‘Apprentice Model’ described by Hoddinott and colleagues [14].

Bridging social capital was also demonstrated through the peer support service being involved in various promotional and awareness-raising activities in their localities. These activities were designed to *‘reach out’* to other women, families as well as wider community members. For example, volunteers were involved in breastfeeding-related community events, e.g. the ‘Milk Run’ [60] and the ‘Big Latch On’ event [61] as well as local Carnival and Baby and Toddler shows. The roles and activities of the volunteers had also featured in local news articles and via radio interviews [62,63]. Furthermore in order to try address some of the wider cultural challenges, educational activities (n = 8, 18 volunteer hours) had been provided at primary schools (4–11 years), high schools (11–16 years) and colleges (16–18 years) to promote positive breastfeeding attitudes and beliefs in the next generation of parents.

While on and off ‘duty’ peer supporters wore their Star Buddies T-shirts in order to promote breastfeeding and their service. In turn, this led to impromptu contacts in terms of peer supporters being approached by mothers, fathers as well as other community members in local and informal settings, e.g. the school playground, shopping centres, leisure clubs, fish and chip shops and by a postman when delivering a parcel; with some 549 contacts of this nature recorded over the evaluation period. The fact that the peer supporters were recognised as a means of support, together with opportunistic opportunities to *‘get information out there to people in lots of different places’* were felt important ‘*to normalise breastfeeding’*. It also meant that women and others could gain guidance and support that they may not have been aware of, or had access to:

*In a supermarket a grandma stood behind me in the queue and she said my daughter in law is having real problems* [. . .] *so I gave her the number of X* [National Breastfeeding Helpline] (PS_Group interview)

These occasions of informal, flexible support were believed to have created a *‘flow’* of information, a *‘ripple’* effect as awareness and access to support resonated across the community.

### Linking social capital

Linking social capital relates to vertical interactions between individuals and people in positions of power. The key difference between linking and bonding/bridging social capital is that it concerns relationships between those who are not necessarily on an equal footing [64,65]. Linking social capital is perceived to be important due to its capacity to connect individuals, and enable access to knowledge and resources to those in authority [64,65]. In this theme we describe how the peer supporters engaged and formed relationships with health, political, community, commercial and statutory sectors to disseminate knowledge and awareness of breastfeeding and peer support, to gain access to women and to promote a positive attitude to breastfeeding throughout the community.

Linking social capital was evident through the peer support service lobbying local politicians and council members for one of their localities to become the first’Breastfeeding Welcome’ town in the UK; with signage to this effect displayed on the town’s perimeter [66]. Vertical relationships were also forged with local businesses to encourage them to become a ‘Breastfeeding Friendly’ establishment. Businesses deemed to be breastfeeding friendly are those where breastfeeding women and young children are welcomed and where women can feel comfortable breastfeeding. Breastfeeding women were subsequently aware of this endorsement through a sticker being visibly displayed in the window of the premises and details listed on a local website. One mother who was initially reticent to feed in public reported how she had been able to overcome her concerns after being told about the *‘cafes, that signed on the breastfeeding support’* and noted how in those places *‘they’re quite happy for you go and do it.* [breastfeeding]*’* As breastfeeding in public has been identified as a key barrier to breastfeeding continuation [67] these links provided important resources for breastfeeding women, as well as being perceived to help ‘*promote a breastfeeding culture*’.

Volunteers also identified how they wore their uniform when accessing professionals for their own healthcare needs e.g. dentist, General Practitioner. They considered how this had enabled promotion and *‘cascading’* of information across these professional groups as well as to wider community members; *‘chemist keeps telling me how she tells people about who I am and where we are’*.

*So I saw my doctor as a personal thing for me and she said, “Oh you do something around breastfeeding don’t you?” So I don’t know whether, again, that makes any difference in her other role, but maybe a mum goes to her and says, oh I’m finding it hard and she might go, oh well I know that there’s a group.* (PS_6)

The peer support service had formed links and relationships with those in authority (e.g. maternity and early years staff) through regular meetings, multi-agency workshops and update events. These opportunities had subsequently enabled the service to gain access to statutory and more formal activities and structures of women-centred support. Examples within the monthly reports concerned volunteers running an infant feeding session as part of the midwifery led antenatal education classes within selected areas (n = 89 sessions delivered). Volunteers also worked alongside a range of statutory and informal professional-run activities and groups, i.e. baby clinics, antenatal clinics, baby groups, lactation consultant-led breastfeeding group, young mother’s groups, toddler groups, baby massage groups and weaning talks. The peer supporters also held or facilitated breastfeeding events within the Children’s Centres, e.g. during National Breastfeeding Week, at Halloween, Harvest, Christmas and Mother’s Day. Overall a total of 378 sessions/events of this nature were provided over the evaluation period, supported by 1,629 volunteer hours. All health/community professionals had been provided with the mobile numbers of the VCs for referral/contact purposes, with 269 health and 88 community professional referrals received during the evaluation period.

As reflected by Hofmeyer & Marck, the exchanges of information and resources as well as efforts at cooperation, coordination, and mutual assistance between professionals and the peer support service meant that women were able to proactively access information and support via multiple locations and opportunities [25]. Furthermore, women were able to find out about the peer support service from different health and community practitioners, through written material developed in conjunction with professionals as well as via personal contacts with other mothers and peer supporters: *‘I phoned them actually because I got the telephone number off a friend’*.

Chang et al. reflects on how social capital encompasses social interaction, trust and shared vision, which subsequently forms the preconditions for knowledge sharing [68]. The on-going contact with professionals across the geographical area was perceived as invaluable in terms of enabling professionals to *‘recognise what we* [peer support service] *do and having more respect for it’*:

*I think at first the health visitors were the hardest but now they’re great. Because it’s showing them how you can help them as well, that you’re there to support them, that’s what it’s about.* (PS_2)

The volunteers would often re-contact the referring professional after contact had been made with the woman. These feedback sessions were believed to be important to provide re-assurance that contact had been made and to raise awareness of the capabilities of the service:

*I had one on Friday that came through, went out on Friday night to see the mum, baby with tongue-tie, referred her to tongue-tie clinic, phoned the health visitor back which is a health visitor I had never dealt with before and told her what had happened, what I’d seen and that I had referred the lady through already and she was like “oh my gosh that’s great have you done that, do I not have to do anything”. Sometimes, the health visitors and midwives don’t know we can do stuff like that.* (PS_Group Interview)

The social interactions through professionals observing women-peer interactions (e.g. at clinics or during home visits) and ‘*free flow of information’* between the peers-professionals was believed to have enhanced the professionals’ confidence and trust in the service. A number of the professionals considered peer support to be an ‘*integral part of my practise’*:

*And, of course, if I was to look at people like X* [VC]*, I just absolutely know that she’s going to be there. I can ring her, she’s always supportive of me, she’ll ring back, she’ll feedback and I know that my client’s going to get a really good service. So I can’t wish for more really.* (HP_5)

Linking social capital in terms of enabling additional knowledge and resources from those in authority [64,65**]**, was also evident through the volunteers making referrals or sign-posting women into other professional-based or specialist services, e.g. fire brigade, benefits advice, birth afterthoughts, speech therapists and tongue-tie clinics:

*I instantly got on to the sign language and they got lessons for her and its things like that. Fire, safety in the home, we do that, get the fire brigade round, link that in*. (PS_2)

It is important to note however that linking social capital was not without its challenges. There were occasions where some professionals raised concerns about a perceived ‘*lack of communication’* with the peer support service and how information was *‘not flowing very well’* in regard to individual cases:

*I think the general thing is that there is no liaison going on at all. The peer supporters, the way it works, have a good relationship with the mother, which is good. But when there are difficulties with feeding, that’s when it would be really helpful to have the good communication and information sharing.* (HP_11)

In turn, these communication difficulties appeared to lead to *‘insecurity’* amongst some of the professionals, together with a desire to *‘find smoother ways of working together’.* A few of the professionals also highlighted tensions in inconsistent advice across the peer-health-community professionals and the potential negative impact on women’s self-efficacy to breastfeed:

*One mum in particular reported so much conflicting advice between different midwives, the peer supporter and then myself. So she said, “look, you know, I’ve been told several different things, I just want to know which one’s right”.* (HP_15)

Some peer supporters expressed concerns about ‘*step* [ping] *on other peoples* [professionals] *toes’* whilst supporting mothers. While these boundaries were often successfully negotiated, in other situations ‘*tension between what the Star Buddies, midwives and health visitors are doing’* was evident*.* This issue tended to occur when there was an overlap in service ‘*you don’t want two* [Star Buddy and professional) *people going in supporting’* and concerns that women would feel *‘bombarded’*. Some professionals also felt that the agenda of the peer supporters was different to their own. Concerns were raised that as peer supporters were ‘*limited to promoting breastfeeding’*, this could be internalised by women as ‘*pressure’*. The peer supporters perceived focus on ‘exclusive’ breastfeeding irrespective of weight gain was also believed to be at odds with the professionals *‘overall health’* agenda:

*There’s been a few instances where really the babies have needed a top up as well because they’ve lost so much weight, but they haven’t been advised to do that because, obviously, it’s not really down to the peer supporters to do that.* (HP_6)

## Discussion

In this paper, we have described how a voluntary model of peer support used bonding, bridging and linking forms of social capital. The findings highlight how horizontal and vertical relationships enabled knowledge sharing, reciprocity, collaboration and trust to facilitate access to women, other community members and to help promote a breastfeeding culture. It is important to consider that bonding, bridging and linking forms of social capital are not mutually exclusive [57,69]. In this peer support service they appeared to operate in synergy. For example, through bridging and linking the peer supporters were able to gain access to, and form ‘bonds’ with women; as well as how ‘bonds’ formed with women and professionals subsequently enabled links and connections to be forged with wider community members. The findings of this study are supported by previous literature in terms of the nature and importance of peer-woman and mother-to-mother relationships [9,70-72], and the value as well as tensions that can exist in peer-professional relationships [4,73-78]. To our knowledge this is the first study that has specifically considered how breastfeeding peer support service can build social capital in a community. It emphasises how relationship building and engagement with women, wider community members, statutory and community professionals as well as commercial and political sectors can enable the dissemination of positive and flexible messages and support for breastfeeding.

This study is focused on a particular model of peer support, in one geographical region in North-West England. While this limits the generalizability of the findings, it does offer insights into how a peer support service can be operationalised. While social capital has become a widely used concept in a range of fields including health for several decades [43], there are on-going critiques of its value as an analytical tool. For some critics the concept of social capital remains rather vague and broad [69] and lacks sufficient terminological precision and theoretical rigour [79]. However, using social capital concepts as a theoretical lens enabled us to illuminate the interplay between peer supporters and structural forces in the community to promote, advocate and maximise reach for breastfeeding support. A broad range of perspectives were included in this study, however the fact that only women who were using the peer support service were recruited limits insights into barriers or access difficulties that other women may face. In this paper it was not the intention to ‘measure’ social capital through an understanding of the relative strength of families and communities. However quantitative methods to determine how and to what extent networks have been developed [80,81] as a result of breastfeeding peer support provision could be incorporated in future research. While women were actively recruited from areas of low and high deprivation via the peer support service, the socio-economic status of women was not collected as part of this study. As demographics such as education and income level are important in determining whether a woman breastfeeds [58] this needs to be addressed within future studies.

Bonding social capital is believed to facilitate reciprocity and solidarity [56,82]. Theoretical insights from the social capital literature have also identified how bonds among community members in turn empowers them to pursue collective interests as they engage with external institutions and organisations [55]. In this study, the bonds formed within the peer support service created collective action through the engagement of health and community professionals, lobbying of local politicians and counsellors, providing education sessions and sign-up of local businesses to encourage public breastfeeding. The bonds created amongst peers and mothers, and subsequently the mother-to-mother relationships in the breastfeeding groups were also perceived to be invaluable in terms of creating friendships, providing reassurance, social support, mutuality, knowledge sharing and normalisation of affects [9,83]. As reflected by others, the breastfeeding group context appeared to create a privileged social space where participants could narrate and potentially reframe personal stories through interacting with their own ‘normative narrative community’ ([84], p.239). The fact that women created social opportunities outside of the breastfeeding groups is also indicative of how the service enabled enhanced social support with suggested benefits in terms of increasing resilience, overall health and well-being [85,86]. Furthermore, as a number of the women wanted to, or were currently accessing peer support training, this emphasises the reciprocity engendered by the service in terms of giving and not just receiving support [87].

While horizontal, ‘in group’ ties provide identity and purpose, it is argued there is a danger that these relationships become too narrow and may exclude individuals [25,69,88]. Furthermore, while linking social capital is perceived to be the weakest form of social capital, it is associated with the most valuable outcomes through access and connection to power structures [54]. Linking, together with bridging capital are also considered essential in order to diffuse information and develop new perspectives [54]. In this study, the peer supporters were identified to ‘bridge’ with vulnerable population groups who are known to be those who are less likely to breastfeed, to recruit volunteers from different ethnic backgrounds and to target support to wider family members, such as partners, and grandparents. However, limitations in reach to certain ethnic groups were identified. The peer supporters’ adornment of a uniform and engagement in community events enabled them to inclusively and flexibly disseminate and provide information and support across the community members. The ‘links’ with those in authority also enabled collaboration, coordination and exchanges of information and resources between professionals and the peer supporters, which subsequently meant that women and others (i.e. family members) were able to obtain information or support in multiple locations and settings as well as gain access to more specialist forms of support.

Relationships built on trust are considered to increase an individual’s willingness to provide useful knowledge to others [89,90]. This was evident in the peer support service through health and community professional’s referrals into the service, with trust and confidence being most evident when there was clear two-way communication. However, the possibility of negative consequences of social capital has been raised by authors such as Hofmeyer & Marck [25], Wakefield & Poland [88] and Portes & Landholt [91]. These authors highlight how social capital can act as a form of social control which may foster self-interest, intolerance, and conformity. In the current study some of the health professionals considered how the peer supporters focus on exclusive identities (e.g. exclusive breastfeeding) limited the personal freedom for women and had potential negative mental health repercussions. However, it is important to emphasise that this perspective appeared contrary to the ethos of the peer support service in terms of supporting the health and well-being of the mother and baby. These insights may well reflect miscommunication or misperceptions between professionals and the peer support service. Alternatively, it may also reflect how professionals operated to serve their own self-interest through the exclusion of this non-professional group. These findings highlight the need for further focused efforts to engage, connect and build peer-professionals relationships in certain areas. This could be achieved through more formalised mentoring relationships between professionals and peer supporters to include co-working, observing each other’s practice and debriefing opportunities [73].

A number of authors consider that further qualitative research is needed to explore the complexities of social capital in context [69,92]. While there has been an over-emphasis on bonding aspects of social capital, more attention to bridging and linking forms of social capital are needed as all three are important for health and well-being [57,69]. We would contend that a similar stance is required in regard to breastfeeding peer support provision. While insights into effectiveness and value are important, equal attention should be paid to how peer supporters can engage between and within different networks and community members in different contexts to embed its service, extend the reach for breastfeeding peer support and to specifically consider how these efforts may help address cultural and social norms associated with infant feeding.

## Conclusion

This study highlights how breastfeeding peer support can build social capital using a synergy of bonding, bridging and linking with community members. Horizontal and vertical relationships between individuals, communities and across public, private and statutory sectors can help to embed peer support into care pathways, may help to promote positive attitudes to breastfeeding and maximise reach for breastfeeding support across the community. However, more focused efforts to engage women from different cultural ethnic backgrounds and address tensions between professionals and peer supporters are warranted. While research into the effectiveness and value of breastfeeding peer support is important, further consideration of all these forms of social capital and their mechanisms are needed. This will enable a greater understanding into how such services can be operationalized within specific cultural and social contexts and wider structures to maximise their potential positive effects.

## Consent

Written informed consent was obtained from all the participants/peer supporters for the publication of this report and any accompanying images.
